# Recommendations for the treatment of vulvar cancer in settings with limited resources: Report from the International Gynecological Cancer Society consensus meeting

**DOI:** 10.3389/fonc.2022.928568

**Published:** 2022-09-20

**Authors:** Fernando Cotait Maluf, Graziela Dal Molin Zibetti, Eduardo Paulino, Andreia Cristina de Melo, Douglas Racy, Robson Ferrigno, Pedro Luiz Serrano Uson Junior, Reitan Ribeiro, Renato Moretti, Jose Carlos Sadalla, Angelica Nogueira Rodrigues, Filomena Marino Carvalho, Glauco Baiocchi, Donato Callegaro-Filho, Roberto Angioli

**Affiliations:** ^1^ Hospital BP Mirante, São Paulo, Brazil; ^2^ Hospital Israelita Albert Einstein, São Paulo, Brazil; ^3^ Oncomed, Rio de Janeiro, Brazil; ^4^ Instituto Nacional de Câncer – INCA, Rio de Janeiro, Brazil; ^5^ Instituto de Oncologia do Paraná, Paraná, Brazil; ^6^ Hospital Sírio Libanês, São Paulo, Brazil; ^7^ Universidade Federal de Minas Gerais, Minas Gerais, Brazil; ^8^ Faculdade de Medicina da Universidade de São Paulo (USP), São Paulo, Brazil; ^9^ A. C. Camargo Cancer Center, São Paulo, Brazil; ^10^ Department of Obstetrics and Gynecology, University Campus Bio-Medico of Rome, Rome, Italy

**Keywords:** vulvar cancer, limited resources, chemotherapy, gynecologic cancers, limited resources countries

## Abstract

**Introduction:**

Due to scant literature and the absence of high-level evidence, the treatment of vulvar cancer is even more challenging in countries facing limited resources, where direct application of international guidelines is difficult. Recommendations from a panel of experts convened to address some of these challenges were developed.

**Methods:**

The panel met in Rio de Janeiro in September 2019 during the International Gynecological Cancer Society congress and was composed of specialists from countries in Africa, Asia, Eastern Europe, Latin America, and the Middle East. The panel addressed 62 questions and provided recommendations for the management of early, locally advanced, recurrent, and/or metastatic vulvar cancer. Consensus was defined as at least 75% of the voting members selecting a particular recommendation, whereas a majority vote was considered when one option garnered between 50.0% and 74.9% of votes. Resource limitation was defined as any issues limiting access to qualified surgeons, contemporary imaging or radiation-oncology techniques, antineoplastic drugs, or funding for the provision of contemporary medical care.

**Results:**

Consensus was reached for nine of 62 (14.5%) questions presented to the panel, whereas a majority vote was reached for 29 (46.7%) additional questions. For the remaining questions, there was considerable heterogeneity in the recommendations.

**Conclusion:**

The development of guidelines focusing on areas of the world facing more severe resource limitations may improve medical practice and patient care.

## Introduction

Squamous-cell carcinoma (SCC) of the vulva accounts for approximately 4% of new cases of gynecologic malignancies in the US, with slightly over 6000 cases per year (1). Despite this rarity, the incidence of vulvar SCC has reportedly increased over the past decades in Australia, Japan, Europe, and North America, particularly among women aged less than 60 years (2–5). Although epidemiological information on the incidence and mortality from vulvar SCC in developing countries is scarce, data from the International Agency for Research on Cancer show that mortality by vulvar cancer in 2019 was more common in Europe (41.1%), followed by Asia (31.6%), North America (10.1%), Latin America and Caribbean (8.3%), Africa (8%), and Oceania (0.8%) (6). Moreover, the burden of human papillomavirus (HPV)-associated malignancies in developing countries and difficult access to health care are reasons for concern (7). In women aged less than 60 years, vulvar SCC is often associated with HPV infection, whereas in older women, this malignancy is frequently associated with vulvar dystrophies, especially lichen sclerosus (8).

In countries facing limited resources, the quality of cancer care is heterogeneous, and practitioners are often faced with challenges that go beyond those seen in more affluent regions of the world (7). It is well known that the implementation of international guidelines is challenging in countries with resource limitations, given that most of these guidelines come from North America and Western Europe (9–11). One alternative for developing countries is to follow adapted or stratified guidelines from prominent organizations, such as the National Comprehensive Cancer Network (NCCN) (12–14). Another option for these countries is to develop their guidelines and consensus panels, something we have tried to do in the current work. We have taken advantage of a large international meeting to organize a panel that could provide consensus recommendations for topics previously identified as relevant in vulvar cancer in limited-resource settings. This article is part of a series of reports from that consensus meeting, convened under the auspices of the International Gynecological Cancer Society.

## Methods

### Composition, organization, and objectives of the panel

**Figure 1:**
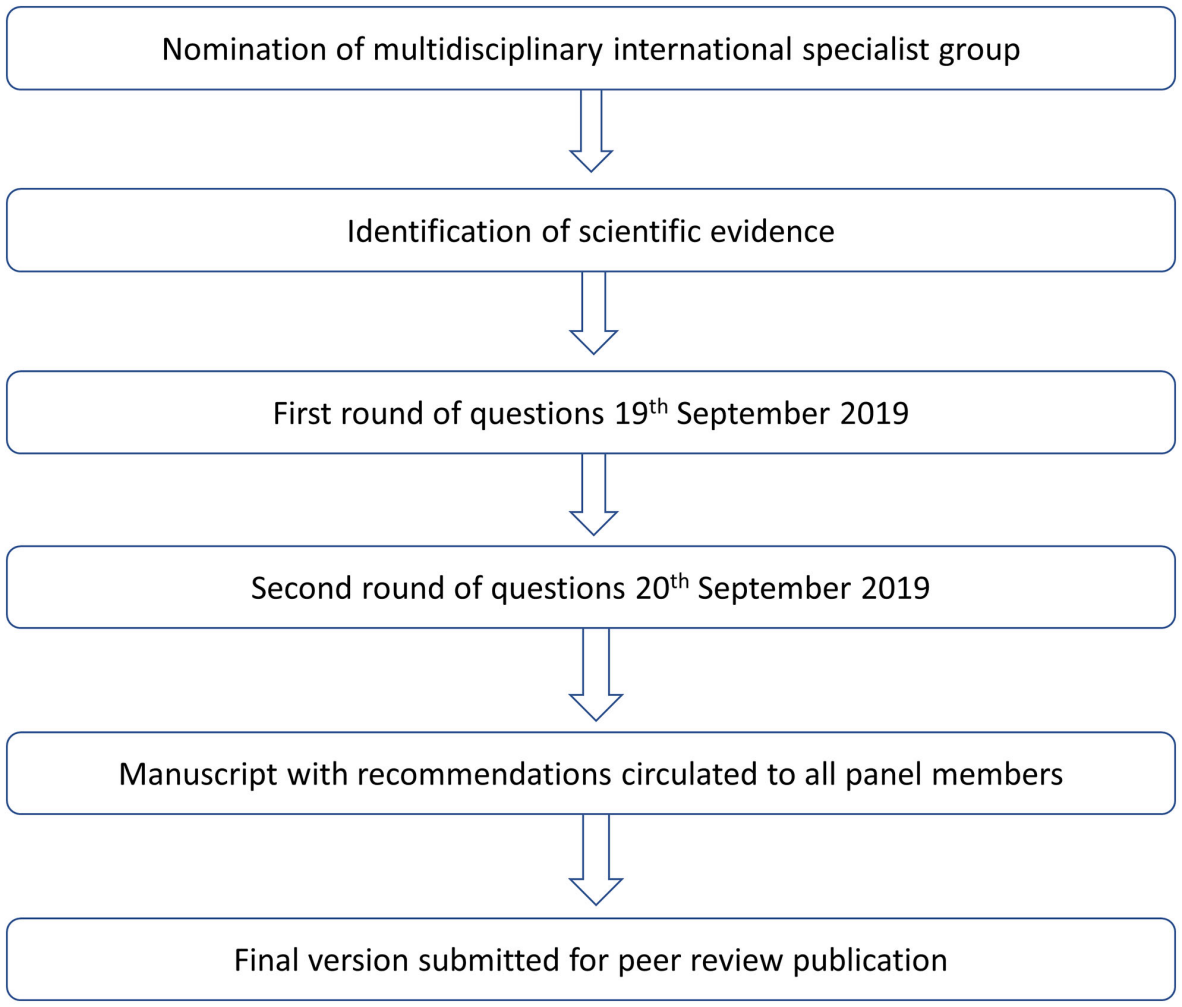
Development process.

The questions addressed by the panel were proposed by a 15-member committee as the most relevant for decision-making in areas facing resource limitations. The panel, composed of invited specialists in gynecological oncology from 38 developing countries in Africa, Asia, Eastern Europe, Latin America, and the Middle East, aimed to provide recommendations on salient issues that affect the management of vulvar cancer in these areas (**Supplementary Table 1**). Panel members were opinion leaders in pathology, gynecology, oncological surgery, medical oncology, radiation oncology, and radiology in their respective counties. Using an electronic voting system, the panel provided answers to the questions in polling sessions held on 19 and 20 September 2019 during the International Gynecological Cancer Society congress in Rio de Janeiro, Brazil **(** **Figure 1** **)**.

One polling session with multiple-choice questions was scheduled for each of the main topics that constitute the subheadings described below. When answering each multiple-choice question, panel members were instructed to consider that their recommended intervention was approved and available, with no contraindications in the scenario described by the corresponding question. Moreover, recommendations were to be given for non-frail patients (defined as having an Eastern Cooperative Oncology Group [ECOG] performance status between 0 and 2) and for patients with squamous cell carcinoma of the vulva. Finally, the staging classification used throughout was the latest one provided by the International Federation of Obstetrics and Gynecology (15).

### Definition of resource limitation

The World Bank classifies country economies into four groups according to their average income: high, upper-middle, lower-middle, and low (16). Even though the panel included members from countries that may belong to different income groups, the socioeconomic framework used herein relates to the availability of ideal resources. This is especially relevant in some of the countries represented, which have heterogeneous healthcare systems. Regardless of the situation in individual countries, the focus of the current work is on “area” rather than “country”, under the assumption that medical practice may not be necessarily constrained in a whole country and still be subject to resource limitation in some of its areas or settings. Finally, resource limitation was broadly defined as limited access to qualified surgeons, contemporary imaging or radiation-oncology techniques, antineoplastic drugs, or overall funding for the provision of state-of-the-art health care.

### Statistical analysis

Results for each of the questions addressed by the panel are presented descriptively and grouped according to clinical setting or issue. If at least 75% of the voting members selected a particular option for a given question, consensus was present. If between 50.0% and 74.9% of the voting members selected a particular option, this was considered as a majority vote, but no consensus. The percentages shown herein do not consider in their denominator the response option “unqualified to answer”, which was available at the meeting. On the other hand, the response option “abstain” was considered in the denominator for each question; this option referred to cases for which a member felt impeded to provide a qualified response for reasons other than lack of knowledge, including the presence of conflicts of interest.

## Results

### Section 1: Staging of vulvar cancer

There was no consensus for any of the three questions related to staging of vulvar cancer in areas with limited resources (**Supplementary Table 2**). Pelvic and abdominal ultrasound and chest X-ray were the set of imaging tools suggested more frequently for staging of tumors ≤2.0 cm in greatest dimension (38.7%), followed closely by thoracic, abdominal, and pelvic computed tomography (CT) or pelvic magnetic resonance imaging (MRI; 28.2%). For tumors >2.0 cm or suspected lymph-node involvement, there was majority vote (66.4%) for the thoracic, abdominal, and pelvic CT (or pelvic MRI). Likewise, there was a majority vote (63.8%) for thoracic, abdominal, and pelvic CT for patients with suspected metastatic vulvar cancer when not all the imaging tools are available.

Recommendation by majority vote:

- For tumors >2.0 cm, suspected lymph-node involvement, or metastatic disease, staging with thoracic CT scans and abdominal CT scans or pelvic MRI is recommended.
- For patients with suspected metastatic vulvar cancer when not all the imaging tools are available, thoracic, abdominal, and pelvic CT is recommended.

### Section 2: Surveillance of vulvar cancer

Even though there was no consensus for the two questions related to surveillance, both had one option with the majority vote (**Supplementary Table 3**). When questioned how often to follow up patients with early-stage vulvar cancer undergoing curative treatment, 57.6% of panel members recommended follow-up every 3 months in the first 2 years, and every 6 months afterward until 5 years from the treatment. Likewise, for patients undergoing curative treatment with early-stage disease, 71.2% of panelists indicated clinical examination as sufficient for follow-up, with 27.3% recommending imaging tests.

Recommendation by majority vote:

- For early-stage vulvar cancer undergoing curative treatment, optimal follow-up should be every three months in the first 2 years, and every 6 months afterward until 5 years from the treatment. Clinical examination should be sufficient for these patients.

### Section 3: Early-stage vulvar cancer

Fifteen questions were asked about early-stage disease (**Supplementary Table 4**). Consensus was present for only three of those questions. For patients with T1b or T2 vulvar carcinomas >2 cm from the midline and positive homolateral inguinal lymph node, contralateral inguinofemoral lymphadenectomy was recommended by 85.4% of panelists as the treatment of choice. For early-stage (I or II), node-negative vulvar cancer after lymphadenectomy, when the tumor is <4 cm and there are negative margins, 82.2% of panelists recommended observation. In institutions where there is only a cobalt machine, most panelists (92.2%) considered that patients with vulvar cancer who need treatment for their pelvic nodes can be treated with primary or adjuvant external radiotherapy.

In addition to the above questions, a majority vote was present for four of 15 questions. For patients with a T1b or T2 vulvar carcinoma less than 4 cm and close to 2 cm from the midline, with unilateral uptake on lymphoscintigraphy, the recommended approach for the contra-lateral side was inguinofemoral lymphadenectomy in 60.4% of cases. In areas with available radiotherapy and patients with a T1b or T2 vulvar carcinoma with more than 2 cm distance from the midline and positive homolateral inguinal lymph node, the recommended treatment for the contralateral inguinal lymph nodes was contralateral inguinofemoral lymphadenectomy in 58.7% of cases, and 10.1% would proceed to radiotherapy alone. If adjuvant radiotherapy alone was the treatment chosen in node-negative cases, 50.0% of the panelists recommended only the primary tumor site (vulva) as the radiation volume. Finally, the treatment recommendation for patients with resected vulvar cancer and macroscopic nodal disease or with >1 micrometastasis-involved nodes after lymphadenectomy was adjuvant concomitant chemoradiation in the opinion of 57.4% of panelists.

For the other eight questions, there was generally considerable heterogeneity in the responses, although in a few cases, two options shared the majority of the votes. For example, the diagnostic choices made for sentinel lymph node dissection in patients with T1b or T2 vulvar carcinoma <4 cm was technetium scintigraphy or blue dye (40.2%) or both (32.7%). Likewise, the treatment recommendation for patients with resected vulvar cancer and one nodal micrometastasis after lymphadenectomy was equally divided (33.0% each between observation and adjuvant radiotherapy alone). In a similar patient in areas where radiotherapy is not available, observation (44.3%) and re-operation with inguinofemoral lymphadenectomy (38.9%) had a nearly similar preference. Finally, when radiotherapy is not available for a patient with resected vulvar cancer and macroscopic nodal disease or with >1 micrometastatic node after lymphadenectomy, panelists were divided between re-operation with inguinofemoral lymphadenectomy (37.1%) and adjuvant chemotherapy alone (46.1%).

Recommendation by consensus:

- For patients with T1b or T2 vulvar carcinomas with >2 cm from the midline and positive homolateral inguinal lymph node, contra-lateral inguinofemoral lymphadenectomy should be recommended.
- For early-stage (I or II), node-negative vulvar cancer after lymphadenectomy, when the tumor is <4 cm and there are negative margins, no adjuvant treatment is necessary.
- In institutions where there is only a cobalt machine, patients with vulvar cancer who need treatment for their pelvic nodes can be treated with primary or adjuvant external radiotherapy.

Recommendation by majority vote:

- For patients with a T1b or T2 vulvar carcinoma <4 cm and close to 2 cm from the midline, with unilateral uptake on lymphoscintigraphy, contralateral inguinofemoral lymphadenectomy should be recommended.
- In areas with radiotherapy available, in patients with a T1b or T2 vulvar carcinoma more than 2 cm distance from the midline and positive homolateral inguinal lymph node, contralateral inguinofemoral lymphadenectomy should be recommended.
- Adjuvant concomitant chemoradiation should be offered for patients with resected vulvar cancer and macroscopic nodal disease or with >1 micrometastasis-involved nodes after lymphadenectomy.

### Section 4: Locally advanced vulvar cancer

Fourteen questions were asked about locally advanced vulvar cancer (**Supplementary Table 5**). Consensus was present for four of those questions. In institutions where there is only conventional, two-dimension (2D) radiotherapy, 95% of panelists agree that patients with vulvar cancer who need treatment for pelvic nodes can be treated, regardless of whether radiation is given with primary or adjuvant intent. Likewise, 94.4% of panelists endorsed the same approach for institutions where there are only cobalt machines. Cisplatin was considered as the best radiosensitizing agent for patients with locally advanced vulvar cancer in areas with limited resources by 86.4% of voters. Finally, concomitant chemoradiation is the treatment of choice for patients with unresectable vulvar cancer in the opinion of 77.4% of panelists.

A majority vote was present for six of 14 questions. In locally advanced disease, 50.0% of panelists considered radical vulvectomy with bilateral inguinofemoral lymphadenectomy followed by radiation as the best treatment when chemotherapy is not available. In patients with locally advanced disease and poor geriatric score and/or poor performance status, the best treatment when radiation therapy is not available is best supportive care according to 55.0% of panelists. For similar patients when chemotherapy is not available, best supportive care is also the best treatment in the opinion of 53.1% of panelists. Radiation volume including vulva and pelvic/inguinal nodes was recommended for patients with pathologic nodal involvement in inguinal or pelvic sites and a resected primary tumor with negative margins according to 67.7% of panelists. The minimal option for patients with vulvar cancer who need external radiotherapy for the treatment of pelvic nodes is 2D radiotherapy in the opinion of 73.6% of panelists. Finally, in patients with locally advanced disease who are ineligible to cisplatin, carboplatin is recommended as the radiosensitizing agent by 68.2% of panelists.

For the remaining four questions, there was more heterogeneity in the responses. In two cases, the numerical variation concerned more similar treatment strategies, which was the case for the order of administration of radical surgery and chemotherapy when radiation therapy is not available for the treatment of locally resectable advanced disease involving the urethra or anus, or between the preference of radiation alone or chemoradiation for the treatment of locally advanced disease in patients with poor geriatric score and/or poor performance status. In the other two cases, the variation concerned more differing approaches. A similar proportion of panelists indicated palliative radiation therapy alone with hypofractionation and best supportive care for patients with unresectable disease and poor geriatric score and/or poor performance status. Likewise, the panel was divided between chemoradiation and primary cytoreductive surgery followed by chemoradiation for patients with bulky inguinal lymph node metastasis in an area without formal training in gynecologic oncology.

Recommendation by consensus:

- Patients with vulvar cancer who need treatment for pelvic nodes can be treated with conventional, two-dimension (2D) radiotherapy, including cobalt technique if there is the only cobalt radiation available, regardless of whether radiation is given with primary or adjuvant intent.
- Cisplatin is considered as the best radiosensitizing agent for patients with locally advanced vulvar cancer.
- Concomitant chemoradiation should be the treatment of choice for patients with unresectable vulvar cancer.

Recommendation by majority vote:

- In locally advanced disease, radical vulvectomy with bilateral inguinofemoral lymphadenectomy followed by radiation
- Best supportive care should be offered for patients with locally advanced disease and poor geriatric score and/or poor performance status when radiation therapy or chemotherapy is not available.
- Radiation volume is recommended for patients with pathologic nodal involvement in inguinal or pelvic sites and a resected primary tumor with negative margins should comprise the vulva and pelvic nodes.
- The minimal option for patients with vulvar cancer who need external radiotherapy for the treatment of pelvic nodes is 2D radiotherapy.
- Carboplatin is recommended as the radiosensitizing agent if the patient is ineligible to cisplatin.

### Section 5: First-line treatment of metastatic or locoregionally recurrent vulvar cancer

There was no consensus for any of the four questions related to first-line treatment of metastatic or locoregionally recurrent vulvar cancer in areas with limited resources (**Supplementary Table 6**). However, there was a majority vote for three of those questions. When asked about the recommended first-line chemotherapy regimen for patients with locoregionally recurrent or metastatic vulvar cancer not amenable to salvage locoregional treatment and no contraindication to cisplatin, panelists’ votes were nearly equally divided into three options: cisplatin plus fluorouracil (35.7%), cisplatin plus paclitaxel (28.6%), and carboplatin plus paclitaxel (35.7%). Concerning the first-line chemotherapy regimen for patients in that same setting, but with contraindication to cisplatin, the recommendation by 66% of the panelists was carboplatin plus paclitaxel. When asked about which non-platinum regimen they recommended as first-line, single-agent paclitaxel was recommended by 58.3% of the panelists, while 8.3% gave preference to gemcitabine; of note, however, nearly 20% of panelists would not recommend a non-platinum agent in this setting. Finally, when asked about their preference for first-line treatment of patients with locoregionally recurrent or metastatic vulvar cancer not amenable to salvage locoregional treatment when access to taxanes is not possible, cisplatin plus 5-FU was the predominant option (54.2%), followed by cisplatin plus gemcitabine (29.2%) and single-agent cisplatin (16.7%).

Recommendation by majority vote:

- In patients with locoregionally recurrent or metastatic vulvar cancer with contra-indication to cisplatin, carboplatin plus paclitaxel is the regimen of choice.
- The non-platinum regimen of choice is single-agent paclitaxel.
- The non-taxane regimen of choice is cisplatin plus fluorouracil.

### Section 6: Locoregionally recurrent, potentially curable vulvar cancer

Consensus as defined here was achieved only for two questions (**Supplementary Table 7**). For the management of potentially resectable local recurrence, without suspicion of lymph node involvement, in patients submitted initially only to surgery and without comorbidities, 80% of panelists recommended salvage surgery alone; likewise, for patients in whom cisplatin could not be recommended as a radiosensitizing agent due to contraindications, 75.8% of panelists opted for carboplatin as part of treatment for patients with local recurrence without suspicion of lymph-node involvement. Moreover, majority of votes were achieved for at least two-thirds of panelists for one option in the following scenarios: (1) for potentially resectable local recurrence, without suspicion of lymph node involvement, in patients submitted initially only to surgery and with comorbidities and/or contra-indication to cisplatin, 71% recommended salvage surgery alone if radiotherapy is not available; (2) likewise, for similar patients submitted initially to radiation therapy, salvage surgery alone (if resectable) was recommended by 67.9% of panelists; (3) for a clinical lymph-node recurrence in a patient without comorbidities treated initially only with surgery, 66.7% indicated salvage surgery (if resectable), followed by cisplatin-based therapy; and (4) for similar patients treated initially with surgery and adjuvant radiation or chemoradiation, the recommendation was salvage surgery (if resectable), followed by carboplatin and paclitaxel in 67.5% of cases. For one question (clinical lymph-node recurrence in a patient with comorbidities and/or contra-indication to cisplatin-treated initially only with surgery when radiotherapy is available), a majority vote (54.3%) was obtained for salvage surgery (if resectable), followed by weekly cisplatin 40 mg/m² IV with radiation therapy. For the other six questions, none of the options had at least 50% of the votes.

Recommendation by consensus:

- Salvage surgery should be recommended for the management of potentially resectable local recurrence, without suspicion of lymph node involvement, in patients submitted initially to surgery and without comorbidities.
- Carboplatin is recommended as the radiosensitizing agent if the patient is ineligible to cisplatin.

Recommendation by majority vote:

- For potentially resectable local recurrence, without suspicion of lymph node involvement, in patients with comorbidities, contraindication to cisplatin, and without available radiotherapy, salvage surgery is recommended.
- For potentially resectable local recurrence, without suspicion of lymph node involvement, in patients submitted initially to radiation therapy, salvage surgery is recommended.
- For a clinical lymph-node recurrence in a patient without comorbidities treated initially only with surgery, salvage surgery (if resectable) followed by cisplatin-based therapy is recommended.
- For a clinical lymph-node recurrence in patients treated initially with surgery and adjuvant radiation or chemoradiation, the recommendation is salvage surgery (if resectable), followed by carboplatin and paclitaxel.
- For a clinical lymph-node recurrence in a patient with comorbidities and/or contraindication to cisplatin, treated initially only with surgery when radiotherapy is available, salvage surgery is recommended, followed by radiation therapy.

### Section 7: Subsequent lines and drugs used in vulvar cancer

As shown in **Supplementary Table 8**, consensus was not reached for any of the questions related to vulvar cancer failing platinum-based therapy, previously treated disease when no clinical trial is available, or treatment for oligometastatic (<4 lesions and restricted to one organ) advanced vulvar cancer. However, a majority vote was obtained for two of the four questions: (1) 72.1% of panelists recommended best supportive care for patients with performance status ≥*2*, regardless of line of treatment, in metastatic vulvar cancer previously treated when no clinical trial is available; and (2) the second-line choice for patients who failed platinum-based therapy was paclitaxel in 60% of cases. Of note, there was considerable heterogeneity in the approach to oligometastatic disease. About drugs used in vulvar cancer included in the World Health Organization (WHO) essential medicines list that can be purchased at an affordable price from generic manufacturers, only paclitaxel, 5-FU and gemcitabine were considered as appropriate treatment options for women with metastatic vulvar cancer by at least 75% of panelists (**Supplementary Table 9**).

Recommendation by majority vote:

- Best supportive care is recommended for patients with performance status ≥*2*, regardless of line of treatment, in metastatic vulvar cancer previously treated when no clinical trial is available.
- Paclitaxel is the second-line regimen of choice after platinum-based chemotherapy.

## Discussion

To the best of our knowledge, this is the first consensus meeting and the first attempt to provide recommendations for vulvar cancer in a large number of countries with considerable resource limitations and a heterogeneous healthcare system. Even though consensus for a larger number of questions would be a desirable feature of this initiative, consensus was reached for nine of 62 (14.5%) questions presented to the panel; moreover, a majority vote was present for 29 (46.7%) additional questions related to limited-resource areas. In early-stage disease, consensus was reached in the following cases: (1) when radiotherapy is not available, contra-lateral inguinofemoral lymphadenectomy is recommended for patients with T1b or T2 vulvar carcinomas >2 cm from the midline and positive homolateral inguinal lymph node; (2) observation is recommended for stage I or II, node-negative vulvar cancer <4 cm, and with negative margins after lymphadenectomy; (3) cobalt is an acceptable radiation modality. In locally advanced disease, consensus was reached for the following questions: (1) 2D radiotherapy is an acceptable radiation modality; (2) telecobalt is an acceptable radiation modality; (3) cisplatin is the best radiosensitizing agent; (4) chemoradiation is the treatment of choice for patients with unresectable vulvar cancer. In locoregional recurrent disease, consensus was reached in the following cases: (1) for potentially resectable local recurrence, salvage surgery should be recommended; (2) in patients ineligible to cisplatin, carboplatin should be recommended.

Considering the staging, the combination of ultrasound and fine-needle aspiration cytology of groin nodes provides a sensitive and specific tool for preoperative assessment in selected patients with vulvar cancer. Ultrasound is easily available even in countries with limited resources and has lower costs compared with other imaging methods (17–19).

Radiation therapy plays a key role in the treatment of early and locally advanced vulvar cancer (20). However, in several countries facing resource limitations, widespread availability of contemporary radiation therapy techniques is often a hindrance to state-of-the-art oncology care (21). Probably as a reflection of the current situation, panelists almost unanimously considered that cobalt machines are sufficient for the treatment of patients with early or locally advanced vulvar cancer when more advanced modalities are not available (20). Adjuvant radiotherapy alone was the recommendation by 38.7% of panelists, and 50.0% recommended irradiation of the primary tumor site. In resectable, locally advanced disease involving the urethra or anus, when radiotherapy is not available, the preferred option by the panel was chemotherapy followed by surgery, even though no majority vote was reached. In this difficult situation for which chemoradiation is the preferred option (22), the unavailability of radiotherapy is clearly a major obstacle to adequate care in these world areas. When chemotherapy is not available, a less likely scenario, there is no sufficient evidence to support recommendations, something that may underlie the majority vote (50.0%) for radical surgery followed by radiation. On the other hand, carboplatin was favored by the panel for patients ineligible to cisplatin. Finally, the consensus on chemoradiation for patients with unresectable vulvar cancer is supported by observational studies and a single-arm trial (23, 24).

Regarding the radiation volume for patients with vulvar cancer presenting pathologic node involvement in inguinal or pelvic sites after margin-negative resection of the primary, 67.7% of voters recommended the inclusion of the vulva and inguinal and pelvic nodes. In these cases, radiation should be delivered to the inguinal, external iliac, internal iliac, and obturator regions bilaterally, and several institutions include the vulva to reduce the risk of recurrence, particularly when there are other risk factors (such as close margins, extensive lymph vascular invasion, or in-transit lesions).

There was no consensus for the questions related to locally advanced disease among patients with poor geriatric score and/or poor performance status, and the recommendations with the largest proportion of votes typically involved radiation alone or chemoradiation. Indeed, there is some literature support for radiation alone among patients unable to tolerate the combined approach (25–27). For similar patients with no access to radiotherapy, there was a majority vote for best supportive care (55.0%), and chemotherapy alone was recommended by 17.6% of voters. Finally, for frail patients with unresectable disease, panel responses were very heterogeneous, reflecting the scarce literature and difficulty related to this situation for which isolated radiation, chemoradiation, and best supportive care are acceptable modalities that should be very carefully individualized.

Another difficult situation likely to be found in areas facing more severe resource limitations is the treatment of patients with bulky inguinal lymph node metastasis when formal training in gynecologic oncology is absent. Despite the scarcity of information on vulvar cancer in this situation, there is evidence from other gynecological malignancies that surgical training is associated with better prognosis when there is a need for complex, systematic lymphadenectomy (28). This likely underlies the panel preference for chemoradiation.

Consensus was reached for only two of the 28 questions related to recurrent and/or metastatic vulvar cancer in areas with limited resources. The practice in this setting is largely dictated by the results of phase 2 trials and extrapolation of cervical cancer data. This probably underlies the similar distribution of choices among three options (cisplatin plus 5-FU, cisplatin plus paclitaxel, and carboplatin plus paclitaxel) by the panel. In settings facing more severe limitations, the first of these is probably the most reasonable, but others are acceptable, and phase 2 trials support the combination of carboplatin and paclitaxel, especially if cisplatin is not an option (29–31). It is noteworthy that carboplatin plus paclitaxel was preferred by nearly a third of panel members even for patients with no contraindication to cisplatin. Moreover, paclitaxel is active as a single agent and is often available in areas with limited resources (32). On the other hand, if taxanes are not available, the use of cisplatin plus 5-FU can probably be justified based on its activity in locally advanced disease, in combination with radiotherapy (29), as well as its low price and widespread availability even in developing countries.

There are scarce data on the role of salvage surgery in recurrent vulvar cancer; moreover, this can be a highly specialized procedure, not often available in areas facing limitations in terms of trained surgeons and infrastructure (33, 34). Despite that, there was consensus, or at least a majority vote, on the role of salvage surgery in settings where radiotherapy is not available, and different options involving salvage surgery were collectively the most frequent choices by the panel, even if radiation is available. The justification for the use of radiotherapy in areas where this modality is available is its role in terms of local control in earlier settings (35); arguably, its effects may be improved by combining chemotherapy, with rates of complete response ranging from 34% to 89% in different settings (36, 37). When cisplatin is contraindicated, there was consensus that carboplatin is the agent of choice, even though it might be inferior to cisplatin (38).

Among patients with local recurrence treated initially with radiation, surgery is the modality of choice; despite its curative potential, its adequate implementation may be challenging in areas facing resource limitations (34). Pelvic exenteration has been associated with a relatively high rate of perioperative complications (39) and should only be performed where there is adequate training and hospital infrastructure.

In the case of patients with a clinical lymph node recurrence, with or without comorbidities, treated initially only with surgery, the panel generally indicated surgical resection as the preferred modality, regardless of whether radiotherapy is available. Of note, surgical expertise and the required infrastructure are often lacking in emerging countries (40–43). Likewise, the availability of radiation therapy facilities varies widely across the globe and may be a problem in several areas represented by this panel (40, 44). In this setting, patients with no previous groin dissection should undergo complete inguinofemoral lymphadenectomy; when available, pre-operative magnetic resonance imaging is useful to delineate the plane of resectability between the nodal mass and the femoral vessels (34). More specifically for patients with no comorbidities, chemoradiation is an option for 28.6% of panelists, likely based on results observed in other settings (36, 45). Likewise, some authors have recommended chemoradiation after resection when the situation allows, noting that the effect of radiation can be optimized by resection of the affected nodes, as radiation sterilizes microscopic disease more successfully than macroscopic disease (34). For patients initially treated with surgery and adjuvant radiation or chemoradiation, surgical salvage remains the modality of choice, as indicated by the panel, although surgery in this setting carries significant morbidity, particularly among patients with gross recurrence (46). For a clinical lymph-node recurrence in a patient without comorbidities treated initially only with surgery, majority of the panel voted in favor of salvage surgery and adjuvant chemotherapy treatment. However, it is important to note that based on the literature nowadays it is not clear that adjuvant chemotherapy in this scenario would improve outcomes.

Regarding non-platinum agents and those to be used in salvage settings, the panel generally expressed a preference for paclitaxel. Even though the literature that supports that this preference remains scant, the agent is usually available in areas facing resource limitation, and is also well-tolerated (32, 47, 48). Gemcitabine is also an option for many panelists, even though case reports have failed to show promising results (48). Finally, 5-FU was also frequently indicated as an option, probably due to its wide availability and tolerance, and despite the absence of solid evidence for its efficacy in very advanced settings (47).

This seems to be the first consensus meeting and the first attempt to provide recommendations for early, locally advanced, and recurrent or metastatic vulvar cancer, involving specialists from many countries with more severe resource limitations. Given that most international guidelines come from North America and Western Europe, it is well known that the implementation of such guidelines is challenging in countries with resource limitations or unique healthcare landscapes (9, 10). One alternative for those countries is to follow adapted or stratified guidelines from prominent organizations, such as the National Comprehensive Cancer Network (NCCN) (12). Although there was considerable heterogeneity in recommendations, we believe these guidelines may help to improve medical practice and patient care in areas of the world facing more severe resource limitations.

## Data availability statement

The original contributions presented in the study are included in the article/**Supplementary Material**. Further inquiries can be directed to the corresponding author.

## Author contributions

FM, GM, EP, AM, DR, RF, PU, RR, RM, JS, AR, FC, GB, DC-F, RA have done substantial contributions to the conception and design of the work. All authors participated in the acquisition, analysis and interpretation of data for the work. All authors participated in the drafting of the work. All authors provide approval for publication of the content. All authors contributed to the article and approved the submitted version.

## Conflict of interest

The authors declare that the research was conducted in the absence of any commercial or financial relationships that could be construed as a potential conflict of interest.

## Publisher’s note

All claims expressed in this article are solely those of the authors and do not necessarily represent those of their affiliated organizations, or those of the publisher, the editors and the reviewers. Any product that may be evaluated in this article, or claim that may be made by its manufacturer, is not guaranteed or endorsed by the publisher.

## Supplementary material

Supplementary Table 1 List of 38 countries represented by the panel.

Supplementary Table 2 Questions related to staging of vulvar cancer.

Supplementary Table 3 Questions related to surveillance of vulvar cancer.

Supplementary Table 4 Questions related to the treatment of early-stage vulvar cancer.

Supplementary Table 5 Questions related to the treatment of locally advanced vulvar cancer.

Supplementary Table 6 Questions related to first-line treatment of metastatic or loco-regionally recurrent vulvar cancer.

Supplementary Table 7 Questions related to loco-regionally recurrent, potentially curable vulvar cancer.

Supplementary Table 8 Questions related to subsequent lines in vulvar cancer.

Supplementary Table 9 Questions related to drugs used in vulvar cancer included in the World Health Organization (WHO) essential medicines list that can be purchased at an affordable price from generic manufacturers.
