# Supplementary material for: Recommendations for the treatment of vulvar cancer in settings with limited resources: Report from the International Gynecological Cancer Society consensus meeting
Source: Front Oncol. 2022 Sep 20;12:928568. doi: 10.3389/fonc.2022.928568 (PMC9530794; doi:10.3389/fonc.2022.928568)
Supplement: Supplementary Table 1 — List of 38 countries represented by the panel. [file DataSheet_1.docx]

**Supplementary Materials for “”RECOMMENDATIONS FOR THE TREATMENT OF VULVAR CANCER IN SETTINGS WITH LIMITED RESOURCES: REPORT FROM THE INTERNATIONAL GYNECOLOGICAL CANCER SOCIETY CONSENSUS MEETING ‘’**

**List of colleagues that voted on the vulvar cancer consensus:**

| *Abraham Peedicayil, MD* |
| --- |
| *Adriana Bermudez, MD* |
| *Adriana Castelo C de Moura, MD* |
| *Ailma larre, MD* |
| *Aisha Mustapha, MBBS, MDRM, FMCOG^1^* |
| *Aknar Calabrich, MD^2^* |
| *Alessandro Buda, MD^3^* |
| *Alexandre da Costa, MD* |
| *Alexandre Pupo Nogueira, MD^4^* |
| *Amanda Karani, MD* |
| *Amilcar Barreta, MD^5^* |
| *Ana Carolina de Rezende, MD^7^* |
| *Ana Carolina Falcão, MD^7^* |
| *Ana Carolina Matos de Queiroz, MD^8^* |
| *Ana Cecilia, MD* |
| *Analia Miele, MD* |
| *Andre Campana, MD* |
| *Andrea Massad Ribeiro, MD* |
| *Andrea Paiva Gadelha Guimaraes, MD* |
| *Andres Estremadoiro Vargas, MD* |
| *Angeles Nico, MD^9^* |
| *Areta Agostinho Rodrigues de Souza, MD ^10^* |
| *Asima Mukhopadhyay, MD^11^* |
| *Caetano da Silva Cardial, MD^12^* |
| *Candice Santos, MD* |
| *Celia Maria Pais Viegas, MD^13^* |
| *Cezar Angelo Alfredo Filho, MD* |
| *Cinthia Barbisan, MD* |
| *Cláudia Bessa Pereira Chaves, MD^14^* |
| *Clement Khoury, MD^15^* |
| *Cristiano de Pádua Souza, MD^16^* |
| *Dalva Guedes Arnaud, MD* |
| *David Atallah, MD, MSc^17^* |
| *Delzio Bicalho, MD* |
| *Dércia Idite Changule,MD^18^* |
| *Diama Vale, MD* |
| *Diocésio Alves Pinto de Andrade, MD^19^* |
| *Eloise Allen Marques de Oliveira, MD^20^* |
| *Erlon Gil , MD* |
| *Fábio Rodrigues, MD^21^* |
| *Fernanda Damian , MD* |
| *Fernando Cordero, MD* |
| *Fernando de Souza Nobrega, MD^7^* |
| *Fuat Demirkiran, MD^22^* |
| *Gabriela Norese, MD^24^* |
| *Geovanne Pedro Mauro, MD^25^* |
| *Gilmara Anne da Silva Resende, MD^26^* |
| *Gonzalo Medina Navía, MD ^27^* |
| *Guilherme Bicudo Barbosa, MD* |
| *Guilherme S. Accorsi, MD ^28^* |
| *Gustavo Focchi, MD* |
| *Gustavo Guitmann, MD* |
| *Henrique Helber, MD* |
| *Ian G Bambury MB.BS, DM^30^* |
| *Igor Austin , MD* |
| *Isabel Cristina Chulvis do Val, MD^32^* |
| *Jeancarllo, MD* |
| *Jitendra Pariyar MD^31^* |
| *João Siufi Neto, MD* |
| *Joseph Bernard, Jr, MD^34^* |
| *Juliana Karassawa Helito , MD* |
| *Juliana Martins Pimenta, MD^35^* |
| *Juliano Rodrigues da Cunha, MD^36^* |
| *Julio Lau, MD^37^* |
| *Julio Teixeira , MD* |
| *Junfen Xu, MD^38^* |
| *Jurema Sales , MD* |
| *Ketheryn Almeida, MD* |
| *Larissa Müller Gomes, MD^39^* |
| *Larissa Sandon, MD* |
| *Leandro Santos A. Resende, MD* |
| *Lillian Morgado Leitão, MD* |
| *Lina Maria Caicedo, MD ^40^* |
| *Linda J Rogers, MD^41, 42^* |
| *Lucas Albuquerque, MD* |
| *Lucas Rios Torres , MD* |
| *Luis E. Medina Fernández, MD^43^* |
| *Luiza Maciel, MD* |
| *Lyliana Coutinho Resende Barbosa, MD^44^* |
| *Marcel Davi Loureiro de Melo , MD* |
| *Marcelo Simonsen, MD* |
| *Maria Carolina Szymanski de Toledo, MD* |
| *Maria Del Pilar Estevez Diz, MD^45^* |
| *Maria Eduarda Bittencourt Damasceno, MD* |
| *Maria Jimena Lange, MD^46^* |
| *Mariana Camargo G. Forghieri, MD ^47^* |
| *Marina Vasco , MD* |
| *Mauricio S. Abrao , MD* |
| *Michelle Almeida, MD* |
| *Mila Oliveira , MD* |
| *Monia Hechiche, MD^48^* |
| *Montassar Ghalleb, MD^49^* |
| *Muhieddine Seoud, MD, FACOG, FACS^50^* |
| *Natacha Phoolcharoen, MD* |
| *Ons Kaabia, MD^51^* |
| *Patricia Izetti , MD* |
| *Poliana Albuquerque Signorini, MD ^52^* |
| *Rachele Grazziotin Reisner, MD^53^* |
| *Raquel C.M. Fernandes , MD* |
| *Renato José Affonso Junior, MD^55^* |
| *Ricardo dos Reis, MD^56^* |
| *Ricardo Pedrini Cruz, MD* |
| *Ricardo Rodrigues de Souza , MD* |
| *Rodrigo Alves Pinto, MD^57^* |
| *Rodríguez Yanina Anahi, MD^58^* |
| *Ronaldo Lúcio Rangel Costa , MD* |
| *Rose Anorlu, MD* |
| *Rossana Catão Zampronha, MD* |
| *Samantha Cabral, MD^45^* |
| *Selma Gadria, MD^59^* |
| *Sergio M Lucchini, MD^61^* |
| *Shahana Pervin, MD^62^* |
| *Shalini Rajaram, MD^63^* |
| *Shylasree TS, MD* |
| *Simona Stolnicu, MD^64^* |
| *Suzana Arenhart Pessini, MD^65^* |
| *Tariane Foiato, MD* |
| *Thais de Almeida, MD* |
| *Thaís Xavier Nogueira de Souza^7^* |
| *Thiago Lins Almeida, MD^66^* |
| *Thomas Konney, MD* |
| *Timothy A.O. Oluwasola, MBBS, MSc, MSCI (Northwestern), FWACS, FMCOG, FACS ^67^* |
| *Tomas Pichlik, MD^61^* |
| *Tracey Adams, MD^62^* |
| *Ts Shylasree, MD* |
| *Vandré Cabral Gomes Carneiro, MD^68^* |
| *Vanessa Alvarenga-Bezerra, MD* |
| *Vinicius Canezin Galletto, MD^7^* |
| *Vinotha Thomas, MD* |
| *Volodymyr Artyomenko MD, PhD, DSc(Med)^69^* |
| *Yue Shi, MD* |

Affiliations:

1: Department of Obstetrics and Gynaecology, Ahmadu Bello University/Teaching Hospital, Zaria, Kaduna State, Nigeria

2: Clinica AMO, Brazil

3: Ospedale Michele e Pietro Ferrero, Gynecologic Oncology Division, Verduno, Cuneo, Italy

4: Hospital Sírio Libanês - São Paulo/SP, Brazil

5: Oncologia 22 de Outubro de Mogi-Mirim-SP, Brazil

7: Hospital Israelita Albert Einstein, SP, Brazil

8: Oncocentro Ceará/Fortaleza-CE , Brazil

9: Instituto de Oncología A. Roffo (Universidad de Buenos Aires), Argentina

10: Kora Saúde- Palmas/TO, Brazil

11: Kolkata Gynecology Oncology Trials and Translational Research Group, Chittaranjan National Cancer Institute, Kolkata, India

12: Departamento de ginecologia, FMABC, Brazil

13: Instituto Nacional de Câncer -Inca, RJ, Brazil

14: Division of Clinical Research and Technological Development, Brazilian National Cancer Institute, Rio de Janeiro, Brazil

15: Department of Radiation Oncology, Clemenceau Medical Center, Beirut, Lebanon

16: Hospital de Câncer de Barretos, Brazil

17: Saint Joseph University, Hôtel Dieu de France university Hospital, Beirut, Lebanon

18: Central Hospital of Maputo, Mozambique

19: InORP – Instituto Oncológico de Ribeirão Preto, Brazil

20: Serviço de Ginecologia e Mama - HOSPITAL ARAÚJO JORGE DA ACCG GOIÁS, Brazil

21: Serviço de Oncoginecolgia e Mastologia do Instituto de Câncer Dr Arnaldo Vieira de Carvalho , SP, Brazil

22: Istanbul university-cerrahpasa gynecologic oncology department Turkey

24: Buenos Aires University Hospital, Argentina.

25: Instituto de Radioterapia do ABC, SP, Brazil

26: Fundação centro de controle de oncologia do Amazonas – FCECON, Brazil

27: Hospital San Juan De Dios Tarija-Bolivia

28: Faculdade de Medicina de Catanduva , Brazil

30: University Hospital of the West Indies, Jamaica

31: Associate Professor & Consultant Gynecologic Oncologist, Civil Service Hospital, Kathmandu, Nepal

32: *Universidade Federal Fluminense, RJ, Brazil*

34: Medical Oncology, Innovating Health International Cancer Program, Port-au-Prince, Haiti

35: Hospital BP Mirante, R Martiniano de Carvalho 965, SP, Brazil

36: Universidade Federal de Uberlândia, MG, Brazil

37: Hospital General San Juan de Dios, Guatemala

38: Department of Gynecologic Oncology, Women’s Hospital, Zhejiang University School of Medicine, Hangzhou 310006, China

39: Centro Paulista de Oncologia - Oncoclínicas/SP, Brazil

40: Gynecologic Oncology , Bogota, Colombia

41: Department of Obstetrics and Gynaecology, Groote Schuur Hospital and the University of Cape Town, Cape Town, South Africa

42: SA MRC/UCT Gynaecological cancer Research Centre

43: Oncology center CEON, Arequipa PERÚ

44: Hospital das Clínicas Samuel Libânio, MG, Brazil

45: Instituto do Cancer do Estado de SP, ICESP, SP, Brazil

46: University Hospital, Buenos Aires, Argentina

47: Dep. Cirurgia de Alta Complexidade do H. M.Maternidade Escola Vila Nova Cachoeirinha, SP, Brazil

*48: Medicine school of Tunis,Tunis Manar University, Tunis Tunisia.*

49: Institute of oncology Tunis, Tunisia

50: Department of Obstetrics and Gynecology, American University Medical Centre, Beirut, Lebanon

51: Université de Sousse, Faculté de Médecine de Sousse, Hôpital Farhat Hached, Service de Gynécologie Obstétrique, 4000, Sousse, Tunisie

52: Fundação Centro de Controle de Oncologia do Amazonas – FCECON, Brazil

53: Instituto Nacional de Câncer -Inca, RJ, Brazil

55: Departamento de radioterapia, Hospital de Base Rio Preto/ Funfarme, Brazil

56: Hospital de Câncer de Barretos, Brazil

57: Oncologia DOR Pernambuco, Brazil

58: Hospital Marie Curie, Hospital Evita Pueblo , Argentina

59: Hannibal Medical Center, Tunisia

61: Gynecology oncology section, Sanatorio Allende de Cordoba, Argentina

62: National Institute of Cancer Research & Hospital , Dhaka, Bangladesh

63: AIIMS Rishikesh, India

64: Department of Pathology, University of Medicine, Pharmacy, Sciences and Technology of Targu Mures, Romania

65: Universidade Federal do Rio Grande do Sul, Brazil

66: Oncoclinicas CPO/PB, Brazil

67: Gynaecologic Oncology Unit, Department of Obstetrics & Gynaecology, College of Medicine, University of Ibadan, and University College Hospital, Ibadan, Nigeria.

68: Hospital de Cancer de Pernambuco, SP, Brazil

69: Honoured Physician of the Ukraine, Ministry of Health of the Ukraine Expert, Department Obstetrics and Gynecology, Odessa National Medical University, Odessa, UKRAINE

**Supplementary Table 1: List of 38 countries represented by the panel**

Argentina, Bahamas, Bangladesh, Bolivia, Brazil, China, Colombia, Egypt, Ethiopia, Georgia, Ghana, Guatemala, Haiti, India, Indonesia, Italy, Jamaica, Jordan, Lebanon, Mexico, Mozambique, Nepal, Nigeria, Pakistan, Peru, Philippines, Qatar, Romania, Saudi Arabia, South Africa, Syria, Thailand, Tunisia, Turkey, Uganda, Ukraine, Venezuela, and Zambia

**Supplementary Table 2.** Questions related to staging of vulvar cancer.

| Questions | **Responses** (%) | | | | | | |
| --- | --- | --- | --- | --- | --- | --- | --- |
| Which imaging tools are indicated for staging of vulvar tumors ≤2.0 cm in greatest dimension in an area with limited resources? | Pelvic and abdominal ultrasound, chest X-ray, and bone scan | Thoracic, abdominal and pelvic CT (or pelvic MRI) and bone scan | Thoracic, abdominal and pelvic CT (or pelvic MRI) | Pelvic and abdominal ultrasound and chest X-ray | None | Abstain | - |
|  | 5.7% | 5.7% | 28.2% | 38.7% | 20.2% | 1.6% | - |
| Which imaging tools are indicated for staging vulvar tumors >2.0 cm and or suspected lymph node involvement in an area with limited resources? | Pelvic and abdominal ultrasound, chest X-ray, and bone scan | Thoracic, abdominal and pelvic CT (or pelvic MRI) and bone scan | Thoracic, abdominal and pelvic CT (or pelvic MRI) | Pelvic and abdominal ultrasound and chest X-ray | Chest X-ray, abdominal and pelvic CT (or pelvic MRI) | None | Abstain |
|  | 1.6% | 6.6% | 66.4% | 10.7% | 12.3% | 1.6% | 0.8% |
| When not all the imaging tools are available, which method is best indicated for systemic staging patients with suspected metastatic vulvar cancer? | Pelvic and abdominal ultrasound, chest X-ray, and bone scan | Thoracic, abdominal and pelvic CT and bone scan | Thoracic, abdominal and pelvic CT | Pelvic and abdominal ultrasound and chest X-ray | None | Abstain | - |
|  | 4.7% | 15.8% | 63.8% | 14.2% | 1.6% | 0% | - |

Legend: Answers to not all questions may total 100% due to rounding.CT, computed tomography; MRI, magnetic resonance imaging.

**Supplementary Table 3. Questions related to surveillance of vulvar cancer.**

| **Question** | **Responses (%)** | | | | | |
| --- | --- | --- | --- | --- | --- | --- |
| How often do you follow up patients treated with early-stage disease after curative treatment in an area with limited resources? | Every 3 months in the first 2 years, after that, every 6 months until 5 years from the treatment | Every 6 months until 5 years from the treatment | Annually until 5 years from the treatment | Every 6 months in the first 2 years, after that, annually until 5 years from the treatment | None | Abstain |
|  | 57.6% | 18.9% | 1.5% | 22.0% | 0% | 0% |
| What image tools do you recommend for follow-up in patients treated with early-stage disease after curative treatment in an area with limited resources? | Clinical examination only | Pelvic and abdominal ultrasound and chest X-ray | Pelvic and abdominal CT and chest X-ray | Abstain | - | - |
|  | 71.2% | 14.4% | 12.9% | 1.5% | - | - |

Legend: Answers to not all questions may total 100% due to rounding.CT, computed tomography.

**Supplementary** **Table 4.** **Questions related to the treatment of early-stage vulvar cancer**.

| **Question** | **Responses (%)** | | | | | |
| --- | --- | --- | --- | --- | --- | --- |
| What is the minimal microscopic margin considered adequate at the final pathology of invasive vulvar carcinoma (T1a – T2)? | Any free margin | 1 mm of free margin | 5 mm of free margin | 8 mm of free margin | 10 mm of free margin | Abstain |
|  | 4.3% | 7.8% | 24.1% | 39.7% | 23.3% | 0.9 |
| For patients with T1b – T2 <4cm vulvar carcinoma, what is the recommended approach regarding the use of sentinel lymph node dissection in an area with limited resources? | Should not be used | Should be used only with technetium, when it is available | Should be used with technetium or blue dye | Should be used with technetium and blue dye | Abstain | - |
|  | 17.7% | 9.4% | 40.2% | 32.7% | 0% | - |
| For patients with T1b – T2 <4cm vulvar carcinoma close to 2 cm from the midline, if the Lymphoscintigraphic shows technetium only in one side, what is the recommended approach on contra-lateral side? | Observation | Inguinofemoral Lymphadenectomy | Use blue dye and sentinel lymph node dissection | Radiotherapy | Abstain | - |
|  | 9.9% | 60.4% | 26.7% | 3.0% | 0% | - |
| Considering patients with T1b or T2 vulvar carcinomas more than 2 cm from the midline and positive homolateral inguinal lymph node, what is the recommended treatment for the contra-lateral inguinal lymph nodes in an area with limited resources? | No need for further treatment | Contra-lateral inguinofemoral lymphadenectomy | No further surgery and proceed with radiation alone | No further surgery and proceed with radiation with chemotherapy | Abstain | - |
|  | 13.7% | 58.7% | 10.1% | 17.4% | 0% | - |
| Considering patients with T1b or T2 vulvar carcinomas more than 2 cm from the midline and positive homolateral inguinal lymph node, what is the recommended treatment for the contra-lateral inguinal lymph nodes, in an area without access to radiotherapy? | No need for further treatment | Contra-lateral inguinofemoral lymphadenectomy | No further surgery and proceed with chemotherapy | Abstain | - | - |
|  | 5.8% | 85.4% | 4.9% | 3.9% | - | - |
| Considering patients with T1b or T2 vulvar carcinomas more than 2 cm from the midline and positive homolateral inguinal lymph node, what is the recommended treatment for the contra-lateral inguinal lymph nodes, in an area where surgeons do not have a full training in gynecology oncology? | No need for further treatment | Contra-lateral inguinofemoral lymphadenectomy | No further surgery and proceed with radiation alone | No further surgery and proceed with radiation with chemotherapy | Abstain | - |
|  | 2.3% | 17.2% | 27.6% | 46.0% | 6.9% | - |
| Considering patients with T1b or T2 vulvar carcinomas more than 2 cm from the midline and positive homolateral inguinal lymph node, what is the recommended treatment for the contra-lateral inguinal lymph nodes, in an area without access to radiotherapy and surgeons do not have a full training in gynecology oncology? | No need for further treatment | Contra-lateral inguinofemoral lymphadenectomy | No further surgery and proceed with chemotherapy | Abstain | - | - |
|  | 8.7% | 24.0% | 40.4% | 26.9% | - | - |
| What is your treatment recommendation for early-stage (I or II) patient with resected vulvar cancer that is node-negative after lymphadenectomy, tumor <4 cm and negative margins in areas with limited resources? | Observation | Adjuvant radiotherapy alone | Adjuvant radiotherapy and chemotherapy | Adjuvant chemotherapy alone | Abstain | - |
|  | 82.2% | 13.9% | 2.0% | 2.0% | 0.0% | - |
| What is your treatment recommendation for early-stage (I or II) patient with resected vulvar cancer that is node-negative after lymphadenectomy, tumor >4 cm or close margins (≤ 8 mm) in areas with limited resources? | Observation | Re-excision | Adjuvant radiotherapy alone | Adjuvant radiotherapy and chemotherapy | Adjuvant chemotherapy alone | Abstain |
|  | 17.1% | 24.3% | 38.7% | 17.1% | 1.8% | 0.9% |
| If adjuvant radiotherapy alone was the treatment chosen in situations above (node-negativecytoreductive), which radiation volume do you recommend in areas with limited resources? | Primary tumor site only (vulva) | Vulva and inguinal nodes | Vulva and inguinal and pelvic nodes | Abstain | - | - |
|  | 50.0% | 27.8% | 16.7% | 5.6% | - | - |
| In institutions where there is only a cobalt machine, can patients with vulvar cancer who need treatment for their pelvic nodes be treated with primary or adjuvant external radiotherapy? | Yes | No | Abstain | Unqualified to answer | - | - |
|  | 92.2% | 6.5% | 1.3% | 0% | - | - |
| What is your treatment recommendation for patients with resected vulvar cancer and one nodal micrometastasis after lymphadenectomy in areas with limited resources? | Observation | Re-operation with inguinofemoral lymphadenectomy | Adjuvant radiotherapy alone | Adjuvant radiotherapy and chemotherapy | Adjuvant chemotherapy alone | Abstain |
|  | 33.0% | 12.0% | 33.0% | 20.0% | 2.0% | 0% |
| In this setting, when radiotherapy is not available, what is your recommendation? | Observation | Re-operation with inguinofemoral lymphadenectomy | Adjuvant chemotherapy alone | Abstain | - | - |
|  | 44.3% | 38.9% | 15.0% | 1.8% | - | - |
| What is your treatment recommendation for patients with resected vulvar cancer and macroscopic nodal disease or with >1 micrometastatic involved nodes after lymphadenectomy in areas with limited resources? | Adjuvant radiotherapy alone | Adjuvant concomitant chemoradiation | Adjuvant chemotherapy alone | Chemotherapy followed by radiotherapy | Radiotherapy followed by chemotherapy | Abstain |
|  | 33.3% | 57.4% | 0.9% | 4.6% | 2.8% | 0.9% |
| In this setting, when radiotherapy is not available, what is your recommendation? | Observation | Re-operation with inguinofemoral lymphadenectomy | Adjuvant chemotherapy alone | Abstain | - | - |
|  | 13.5% | 37.1% | 46.1% | 3.4% | - | - |

Legend: Answers to not all questions may total 100% due to rounding.

**Supplementary** **Table 5.** **Questions related to the treatment of locally-advanced vulvar cancer**.

| **Question** | **Responses (%)** | | | | | | | |
| --- | --- | --- | --- | --- | --- | --- | --- | --- |
| In locally resectable advanced disease, involving urethra or anus, what is the best treatment in an area with limited resources when radiation therapy is not available? | Radical vulvectomy with bilateral inguinofemoral lymphadenectomy alone | Radical vulvectomy with bilateral inguinofemoral lymphadenectomy followed by chemotherapy | Chemotherapy followed by radical vulvectomy and removal of gross nodes | Chemotherapy followed by radical vulvectomy and bilateral inguinofemoral lymphadenectomy | Inguinofemoral lymphadenectomy followed by chemotherapy and vulvectomy | None | Abstain | - |
|  | 10.2% | 20.5% | 11.4% | 37.5% | 5.7% | 8.0% | 6.8% | - |
| In locally advanced disease, what is the best treatment in an area with limited resources when chemotherapy is not available? | Radical vulvectomy with bilateral inguinofemoral lymphadenectomy alone | Radical vulvectomy with bilateral inguinofemoral lymphadenectomy followed by radiation | Radiation followed by radical vulvectomy and removal of gross nodes | Radiation followed by radical vulvectomy and bilateral inguinofemoral lymphadenectomy | Inguinofemoral lymphadenectomy followed by radiation and vulvectomy | Abstain | - | - |
|  | 8.7% | 50.0% | 6.5% | 17.4% | 8.7% | 8.7% | - | - |
| In locally advanced vulvar cancer and poor geriatric score and/or poor performance status, what is the best treatment in an area with limited resources? | Radical vulvectomy with bilateral inguinofemoral lymphadenectomy alone | Radical vulvectomy as a palliative procedure | Chemoradiation followed by radical vulvectomy and removal of gross nodes | Chemoradiation followed by radical vulvectomy and bilateral inguinofemoral lymphadenectomy | Radiation alone | Chemoradiation | Best supportive care | Abstain |
|  | 2.2% | 13.2% | 4.4% | 2.2% | 30.8% | 35.2% | 12.1% | 0% |
| In locally advanced vulvar cancer and poor geriatric score and/or poor performance status, what is the best treatment in an area with limited resources when radiation therapy is not available? | Radical vulvectomy | Radical vulvectomy with bilateral inguinofemoral lymphadenectomy alone | Radical vulvectomy with bilateral inguinofemoral lymphadenectomy followed by chemotherapy | Chemotherapy followed by radical vulvectomy and removal of gross nodes | Chemotherapy followed by radical vulvectomy and bilateral inguinofemoral lymphadenectomy | Chemotherapy alone | Best supportive care | Abstain |
|  | 9.9% | 4.4% | 2.2% | 3.3% | 5.5% | 17.6% | 55% | 2.2% |
| In locally advanced vulvar cancer and poor geriatric score and/or poor performance status, which is the best treatment in an area with limited resources when chemotherapy is not available? | Radical vulvectomy | Radical vulvectomy with bilateral inguinofemoral lymphadenectomy alone | Radical vulvectomy with bilateral inguinofemoral lymphadenectomy followed by radiation | Radiation followed by radical vulvectomy and removal of gross nodes | Radiation followed by radical vulvectomy and bilateral inguinofemoral lymphadenectomy | Inguinofemoral lymphadenectomy followed by radiation and vulvectomy | Best supportive care | Abstain |
|  | 6.2% | 6.2% | 9.9% | 9.9% | 6.2% | 0.0% | 53.1% | 8.6% |
| Which radiation volume do you recommend for patients with vulvar cancer presenting pathologic node involvement in inguinal or pelvic sites and primary resected tumor with negative margins? | Vulva and inguinal nodes | Vulva and inguinal and pelvic nodes | Inguinal and pelvic nodes | Abstain | Unqualified to answer | - | - | - |
|  | 7.4% | 67.7% | 25.0% | 0.0% | 0.0% | - | - | - |
| Which external radiotherapy technique is recommended as a minimal option for patients with vulvar cancer who need treatment for pelvic nodes in areas with limited resources? | Conventional (2D) | Conformal | Abstain | Unqualified to answer | - | - | - | - |
|  | 73.6% | 26.4% | 0% | 0% | - | - | - | - |
| In Institutions where there is only conventional (2D) radiotherapy technique, can patients with vulvar cancer who need treatment for pelvic nodes be treated with primary or adjuvant external radiotherapy? | Yes | No | Abstain | Unqualified to answer | - | - | - | - |
|  | 95.0% | 3.8% | 1.3% | 0% | - | - | - | - |
| In Institutions where there is only a cobalt machine, can patients with vulvar cancer who need treatment for pelvic nodes be treated with primary or adjuvant external radiotherapy? | Yes | No | Abstain | Unqualified to answer | - | - | - | - |
|  | 94.4% | 5.6% | 0% | 0% | - | - | - | - |
| In patients with locally advanced vulvar cancer, what is the best radiosensitizing agent in an area with limited resources? | Cisplatin | Cisplatin and fluorouracil | Carboplatin | Fluorouracil | Gemcitabine | Abstain | - | - |
|  | 83.4% | 10.2% | 3.4% | 0% | 0% | 0% | - | - |
| In patients with locally advanced vulvar cancer and ineligible for cisplatin, which is the best radiosensitizing agent in an area with limited resources? | Carboplatin | Carboplatin and fluorouracil | Fluorouracil | Mitomycin and fluorouracil | Gemcitabine | Taxane | None | Abstain |
|  | 68.2% | 17.7% | 5.9% | 1.2% | 2.4% | 0% | 3.5% | 1.2% |
| What is your treatment recommendation for patients with unresectable vulvar cancer in areas with limited resources? | Palliative radiation therapy alone with hypofractionation | Palliative radiation therapy alone with conventional fractionation | Concomitant chemoradiation | Chemotherapy alone | Best supportive care | Abstain | - | - |
|  | 11.8% | 3.2% | 77.4% | 5.4% | 2.2% | 0% | - | - |
| What is your treatment recommendation for patients with unresectable vulvar cancer with poor geriatric score and/or poor performance status in areas with limited resources? | Palliative radiation therapy alone with hypofractionation | Palliative radiation therapy alone with conventional fractionation | Concomitant chemoradiation | Chemotherapy alone | Best supportive care | Abstain | - | - |
|  | 28.9% | 17.8% | 21.1% | 2.2% | 28.9% | 1.1% | - | - |
| In patients with bulky inguinal lymph node metastasis in an area without formal training in gynecologic oncology, the treatment should include: | Metastatic bulky lymph nodes are indication for chemoradiation, and surgery is not indicated as first approach | If feasible, primary cytorreductive surgery (resection of the macroscopically involved lymph nodes) should be performed before chemoradiation | Systematic inguinofemoral lymphadenectomy should be performed before the treatment with chemoradiation | Radiotherapy alone | Chemotherapy alone | Chemoradiation | Abstain | - |
|  | 20.5% | 24.1% | 7.2% | 1.2% | 1.2% | 43.4% | 2.4% | - |

Legend: Answers to not all questions may total 100% due to rounding.

**Supplementary Table 6.** **Questions related to first-line treatment of metastatic or loco-regionally recurrent vulvar cancer.**

| **Question** | **Answers and frequency of responses** | | | | | | | |
| --- | --- | --- | --- | --- | --- | --- | --- | --- |
| What is the recommended first-line chemotherapy regimen for women with metastatic vulvar cancer or loco-regional recurrence not amenable to salvage loco-regional treatment with no contra-indication to cisplatin in an area with limited resources? | Cisplatin**,** 50 mg/m² IV on day 1 with 5-FU**,** 1000 mg/m² IV on days 1-4 every 3 weeks | Cisplatin, 50 mg/m² IV on day 1 with Paclitaxel**,** 175 mg/m² IV on day 1 every 3 weeks | Cisplatin, 50 mg/m² IV with Gemcitabine**,** 1000 mg/m² IV on days 1, 8 every 3 weeks | Carboplatin AUC 5 and paclitaxel 175 mg/m2 every 3 weeks | Cisplatin, 50 mg/m² IV every 3 weeks | Non-platinum agent alone | Best supportive care | Abstain |
|  | 35.7% | 28.6% | 0% | 35.7% | 0% | 0% | 0% | 0% |
| What is the recommended first-line chemotherapy regimen for women with metastatic vulvar cancer or loco-regional recurrence not amenable to salvage loco-regional treatment with contra-indication to cisplatin in an area with limited resources? | Carboplatin AUC 5 IV on day 1 with 5-FU**,** 1000 mg/m² IV on days 1-4 every 3 weeks | Carboplatin AUC 5 IV on day 1 with Paclitaxel**,** 175 mg/m² IV on day 1 every 3 weeks | Carboplatin AUC 5 IV with Gemcitabine**,** 1000 mg/m² IV on days 1, 8 every 3 weeks | Carboplatin AUC 5 alone IV alone every 3 weeks | Non-platinum agent alone | Best supportive care | Abstain | - |
|  | 16.7% | 66.7% | 0% | 5.6% | 0% | 0% | 11.1% | - |
| If you recommend a non-platinum regimen as first-line, what agent do you recommend in an area with limited resources? | Paclitaxel | Gemcitabine | 5-FU | Ifosfamide | I do not recommend a non-platinum agent as first-line therapy | Abstain | - | - |
|  | 58.3% | 8.3% | 4.2% | 4.2% | 20.8% | 4.2% | - | - |
| What is your first-line treatment for patients with metastatic or recurrent vulvar cancer not amenable to salvage loco-regional treatment in countries without access to taxanes or where taxane-related costs are prohibitive? | Cisplatin and 5-fluorouracil | Cisplatin and gemcitabine | Cisplatin | Gemcitabine | None | Abstain | - | - |
|  | 54.2% | 29.2% | 16.7% | 0% | 0% | 0% | - | - |

Legend: Answers to not all questions may total 100% due to rounding.

**Supplementary Table 7.** **Questions related to loco-regionally recurrent, potentially curable vulvar cancer.**

| **Question** | **Answers and frequency of responses** | | | | | | | |
| --- | --- | --- | --- | --- | --- | --- | --- | --- |
| What is the recommended treatment option for a local recurrence potentially resectable without suspicion of lymph node involvement in a patient who was submitted initially only to surgery and without comorbidities in an area with limited resources where radiotherapy is available? | Weekly cisplatin, 40 mg/m² IV in association with radiation therapy | Radiation therapy alone | Salvage surgery alone | Salvage surgery followed by radiation therapy | Salvage surgery followed by weekly cisplatin, 40 mg/m² IV in association with radiation therapy | Cisplatin-based therapy alone | Best supportive care | Abstain |
|  | 22.6% | 0% | 12.9% | 29.0% | 35.5% | 0% | 0% | 0% |
| What is the recommended treatment option for a local recurrence potentially resectable without suspicion of lymph node involvement in a patient who was submitted initially only to surgery and without comorbidities in an area with limited resources where radiotherapy is not available? | Salvage surgery alone | Platinum-based therapy alone | Platinum-based therapy followed by surgery | Non platinum-based therapy followed by surgery | Best supportive care | Abstain | - | - |
|  | 80.0% | 2.9% | 17.1% | 0% | 0% | 0% | - | - |
| What is the recommended treatment option for a local recurrence potentially resectable without suspicion of lymph node involvement in a patient who was submitted initially only to surgery and with comorbidities and/or contra-indication to cisplatin in an area with limited resources where radiotherapy is available? | Carboplatin combined with radiation therapy | Non-platinum agent with radiation therapy | Radiation therapy alone | Salvage surgery alone | Salvage surgery followed by radiation or chemoradiation therapy | Chemotherapy alone | Best supportive care | Abstain |
|  | 13.9% | 0% | 22.2% | 19.4% | 41.7% | 0% | 0% | 2.8% |
| What is the recommended treatment option for a local recurrence potentially resectable without suspicion of lymph node involvement in a patient who was submitted initially only to surgery and with comorbidities and/or contra-indication to cisplatin in an area with limited resources where radiotherapy is not available? | Salvage surgery alone | Carboplatin-based therapy followed by surgery | Non-platinum based therapy followed by surgery | Carboplatin-based therapy alone | Non-platinum agent alone | Best supportive care | Abstain | - |
|  | 71% | 10.5% | 0% | 2.6% | 2.6% | 10.5% | 2.6% | - |
| What is the recommended treatment option for a local recurrence without suspicion of lymph node involvement in patients with no comorbidities who were submitted to radiation therapy in an area of limited resources? | Weekly cisplatin**,** 40 mg/m² IV with re-radiation therapy | Re-radiation therapy | Salvage surgery alone if resectable | Salvage surgery if resectable, followed by re-radiation therapy | Salvage surgery if resectable, followed by carboplatin and paclitaxel | Cisplatin-based therapy alone | Best supportive care | Abstain |
|  | 5.3% | 0.% | 55.3% | 7.9% | 29.0% | 2.6% | 0% | 0% |
| What is the recommended treatment option for a local recurrence without suspicion of lymph node involvement in patients with comorbidities and/or contra-indication to cisplatin who were submitted to radiation therapy in an area of limited resources? | Carboplatin with re-radiation therapy | Re-radiation therapy | Salvage surgery alone if resectable | Salvage surgery if resectable, followed by re-radiation therapy | Salvage surgery if resectable, followed by carboplatin and paclitaxel | Carboplatin-based therapy alone | Best supportive care | Abstain |
|  | 0% | 0% | 67.9% | 3.6% | 17.9% | 0% | 10.7% | 0% |
| If you do not recommend cisplatin as a radiosensitizing agent with radiation due to contra-indication in this scenario, what would be your choice in an area with limited resources? | Carboplatin | Paclitaxel | Gemcitabine | 5-FU | Abstain | - | - | - |
|  | 75.8% | 3.0% | 3.0% | 15.2% | 3.0% | - | - | - |
| What is the recommended treatment option for a clinical lymph node recurrence in a patient without comorbidities treated initially only with surgery in an area with limited resources where radiotherapy is available? | Weekly cisplatin**,** 40 mg/m² IV with radiation therapy | Radiation therapy alone | Salvage surgery alone if resectable | Salvage surgery if resectable, followed by radiation therapy | Salvage surgery if resectable, followed by weekly cisplatin**,** 40 mg/m² IV with radiation therapy | Cisplatin-based therapy alone | Best supportive care | Abstain |
|  | 28.6% | 0% | 5.7% | 11.4% | 54.3% | 0% | 0% | 0% |
| What is the recommended treatment option for a clinical lymph node recurrence in a patient without comorbidities treated initially only with surgery in an area with limited resources where radiotherapy is not available? | Salvage surgery alone if resectable | Cisplatin-based therapy alone | Salvage surgery if resectable, followed by cisplatin-based therapy | Best supportive care | Abstain | - | - | - |
|  | 19.4% | 8.3% | 66.7% | 5.6% | 0% | - | - | - |
| What is the recommended treatment option for a clinical lymph node recurrence in a patient with comorbidities and/or contra-indication to cisplatin treated initially only with surgery in an area with limited resources where radiotherapy is available? | Carboplatin with radiation therapy | Non-platinum agent with radiation therapy | Radiation therapy alone | Salvage surgery alone if resectable | Salvage surgery if resectable, followed by radiation therapy or chemoradiation | Chemotherapy alone | Best supportive care | Abstain |
|  | 20% | 3.3% | 13.3% | 0% | 60% | 0% | 3.3% | 0% |
| What is the recommended treatment option for a clinical lymph node recurrence in a patient with comorbidities and/or contra-indication to cisplatin treated initially only with surgery in an area with limited resources where radiotherapy is not available? | Salvage surgery alone if resectable | Salvage surgery if resectable, followed by carboplatin-based therapy | Salvage surgery if resectable, followed by non-platinum based therapy | Carboplatin-based therapy followed by surgery if resectable | Carboplatin-based therapy alone | Non-platinum agent alone | Best supportive care | Abstain |
|  | 28.3% | 45.7% | 6.5% | 6.5% | 6.5% | 0% | 6.5% | 0% |
| What is the recommended treatment option for a clinical lymph node recurrence in a patient without comorbidities treated initially with surgery and adjuvant radiation or chemoradiation in an area with limited resources? | Weekly cisplatin with radiation therapy | Re-radiation therapy alone | Salvage surgery alone if resectable | Salvage surgery if resectable, followed by re-radiation therapy | Salvage surgery if resectable, followed by carboplatin and paclitaxel | Cisplatin-based therapy alone | Best supportive care | Abstain |
|  | 2.5% | 0% | 15.0% | 12.5% | 67.5% | 2.5% | 0% | 0% |
| What is the recommended treatment option for a clinical lymph node recurrence in a patient with comorbidities and/or contra-indication to cisplatin treated initially with surgery and adjuvant radiation or chemoradiation in an area with limited resources? | Carboplatin with radiation therapy | Non-platinum agent with radiation therapy | Radiation therapy alone | Salvage surgery alone if resectable | Salvage surgery if resectable, followed by radiation therapy or chemoradiation | Chemotherapy alone | Best supportive care | Abstain |
|  | 2.7% | 0% | 5.4% | 40.5% | 27.0% | 13.5% | 5.4% | 5.4% |

Legend: Answers to not all questions may total 100% due to rounding.CIN, cervical intraepithelial neoplasia; LEEP, loop electrosurgical excision procedure; LLETZ, large loop excision of the transformation zone.

**Supplementary Table 8.** **Questions related to subsequent lines in vulvar cancer.**

| **Question** | **Answers and frequency of responses** | | | | | | | |
| --- | --- | --- | --- | --- | --- | --- | --- | --- |
| What is your second-line choice for patients who failed platinum-based therapy in an area with limited resources? | Paclitaxel | Gemcitabine | 5-FU | Methotrexate | Best supportive care | Abstain | - | - |
|  | 60.0% | 20.0% | 10.0% | 3.3% | 3.3% | 3.3% | - | - |
| What is your third-line treatment choice for patients who failed platinum-based therapy with good performance status in an area with limited resources? | Paclitaxel | Gemcitabine | Vinorelbine | 5-FU | Methotrexate | Best supportive care | Abstain | - |
|  | 17.2% | 37.9% | 0% | 6.9% | 0% | 24.1% | 13.8% | 0.0% |
| For women with metastatic vulvar cancer previously treated and with no clinical trial available, when do you recommend best supportive care in an area with limited resources? | After first-line treatment | After second-line treatment | After third-line or more treatment | P*erformance status* ≥ *2*, unrelated to line of treatment | Abstain | - | - | - |
|  | 9.3% | 14.0% | 4.7% | 72.1% | 0% | - | - | - |
| Would you consider metastasectomy, radiation therapy, or either for oligometastatic (< 4 lesions and restricted to one organ) advanced vulvar cancer (excluding bone metastasis) in an area with limited resources? | In the majority of patients, and I prefer surgery | In the majority of patients, and I prefer radiation | In a minority of patients, and I prefer surgery | In a minority of patients, and I prefer radiation | Both are equivalent, and I consider for the majority of patients | Both are equivalent, and I consider in a minority of patients | I do not recommend either surgery or radiation | Abstain |
|  | 24.6% | 3.5% | 10.5% | 17.5% | 7.0% | 14.0% | 19.3% | 3.5% |

Answers to not all questions may total 100% due to rounding.

**Supplementary Table 9. Questions related to drugs used in vulvar cancer included in the World Health Organization (WHO) essential medicines list that can be purchased at an affordable price from generic manufacturers**.

| **Questions** | **Responses (%)** | | |
| --- | --- | --- | --- |
| Each of the following drugs is on the WHO essential medicines list. You are able to purchase them at an affordable price from generic manufacturers. Which would you consider as appropriate treatment options for women with metastatic vulvar cancer in the setting of limited healthcare resources? | Yes | No | Abstain |
| Ifosfamide | 23.1% | 74.4% | 2.6% |
| Topotecan | 44.4% | 52.8% | 2.8% |
| Paclitaxel | 95.4% | 4.7% | 0% |
| Gemcitabine | 88.2% | 11.8% | 0% |
| Vinorelbine | 40.7% | 51.9% | 7.4% |
| 5-FU | 91.4% | 8.6% | 0.0% |
| Methotrexate | 29.7% | 64.9% | 5.4% |

Legend: Answers to not all questions may total 100% due to rounding.
